# Injectable Anesthesia for Mice: Combined Effects of Dexmedetomidine, Tiletamine-Zolazepam, and Butorphanol

**DOI:** 10.1155/2017/9161040

**Published:** 2017-01-22

**Authors:** Laura A. Cagle, Lisa M. Franzi, Steven E. Epstein, Philip H. Kass, Jerold A. Last, Nicholas J. Kenyon

**Affiliations:** ^1^Division of Pulmonary, Critical Care, and Sleep Medicine, School of Medicine, University of California, Davis, CA, USA; ^2^Department of Surgical and Radiological Sciences, School of Veterinary Medicine, University of California, Davis, CA, USA; ^3^Department of Population Health and Reproduction, School of Veterinary Medicine, University of California, Davis, CA, USA

## Abstract

Anesthetic protocols for murine models are varied within the literature and medetomidine has been implicated in the development of urethral plugs in male mice. Our objective was to evaluate the combination of butorphanol, dexmedetomidine, and tiletamine-zolazepam. A secondary objective was to identify which class of agent was associated with urethral obstructions in male mice. BALB/c male (*n* = 13) and female (*n* = 23) mice were assigned to dexmedetomidine and tiletamine-zolazepam with or without butorphanol or to single agent dexmedetomidine or tiletamine-zolazepam. Anesthesia was achieved in 58% (14/24) of mice without butorphanol and in 100% (24/24) of mice with butorphanol. The combination of dexmedetomidine (0.2 mg/kg), tiletamine-zolazepam (40 mg/kg), and butorphanol (3 mg/kg) resulted in an induction and anesthetic duration of 12 and 143 minutes, respectively. Urethral obstructions occurred in 66% (25/38) of trials in male mice that received dexmedetomidine with a mortality rate of 38% (5/13). Tiletamine-zolazepam, when used alone, resulted in a 0% (0/21) incidence of urethral obstructions. Combination use of dexmedetomidine, tiletamine-zolazepam, and butorphanol results in a longer and more reliable duration of anesthesia than the use of dexmedetomidine and tiletamine-zolazepam alone. Dexmedetomidine is not recommended for use in nonterminal procedures in male mice due to the high incidence of urethral obstructions and resultant high mortality rate.

## 1. Introduction

Optimized injectable and inhalational anesthetic protocols for hours-long anesthesia are essential for laboratory animal research. Many laboratories do not have the ability to use inhalational vapor anesthesia, necessitating the use of injectable anesthetics. Researchers have utilized a wide array of injectable anesthetic protocols including the use of barbiturates, ketamine, or xylazine with varying success [1–9].

The use of dexmedetomidine in conjunction with tiletamine-zolazepam with or without the addition of an opioid as an anesthetic protocol has yet to be evaluated in a mouse model. An opioid receptor agonist can be added to an anesthetic regimen to reduce the dosing of other agents or prolong the duration of anesthesia in some species [10], but we do not know whether this is true in a murine model. Urethral obstructions using medetomidine (combination of levomedetomidine and dexmedetomidine isomer) in combination with ketamine have been documented in male mice [11, 12]. The occurrence of urethral obstructions with the use of medetomidine and ketamine is presumed to be due to the use of medetomidine, as ketamine-xylazine anesthesia has not been associated with this adverse effect [12].

The purpose of this study was to determine whether the addition of an opioid would prolong the anesthetic effects of the combination of dexmedetomidine and tiletamine-zolazepam (Telazol®). A secondary objective was to establish a reliable anesthetic protocol for use in mice by determining appropriate drug dosages. A tertiary objective of this study was to determine which anesthetic agent was associated with urethral obstructions in male mice.

## 2. Animals

Specific pathogen-free BALB/c mice (*n* = 36), 14–25 grams, 4–16-week-old males and females, were obtained from Jackson Laboratory (Bar Harbor, ME) and allowed to acclimatize for one week prior to study enrollment. Animals were housed in plastic cages over autoclaved bedding in a HEPA-filtered laminar flow cage rack on a 12-hour light/dark cycle. All mice were allowed free access to water and a standard diet (Purina Rodent Chow). Procedures with the mice were performed in accordance with an approved IACUC protocol. Mice were routinely screened and cared for by the veterinary staff of the Animal Resource Service at the University of California, Davis, in AAALAC-accredited facilities.

## 3. Materials and Methods

### 3.1. Anesthetic Protocols

Females (*n* = 23) were assigned to receive combinations of dexmedetomidine and tiletamine-zolazepam (DTZ) or combinations of dexmedetomidine, tiletamine-zolazepam, and butorphanol (DTZB) as shown in Table 1. Mice were not assigned to the same dosage group more than once. Dosages in the DTZ group started at 0.4 mg/kg and 20 mg/kg and increased to 0.8 mg/kg and 60 mg/kg, respectively. Dosages in the DTZB group started at 0.2 mg/kg, 10 mg/kg, and 3 mg/kg and increased to 0.6 mg/kg, 40 mg/kg, and 3 mg/kg, respectively. Dexmedetomidine and tiletamine-zolazepam were administered intraperitoneally (IP) while butorphanol was administered via a subcutaneous (SC) injection.

Male mice (*n* = 13) were assigned to groups receiving dexmedetomidine and tiletamine-zolazepam (*n* = 7), dexmedetomidine (*n* = 2), or tiletamine-zolazepam (*n* = 4). Mice were not assigned to the same dosage group more than once. Dexmedetomidine-only animals received dosages ranging from 0.025 to 0.6 mg/kg, while the tiletamine-zolazepam-only groups received dosages ranging from 10 to 80 mg/kg. Combination groups of dexmedetomidine and tiletamine-zolazepam received 0.2 to 0.8 mg/kg and 0 to 50 mg/kg, respectively. Mice were given at least 48 hours between injections and each mouse received multiple injections over a four-week period.

### 3.2. Anesthetic Monitoring and Recovery

Mice were placed on warming pads and monitored continuously, with assessment of heart rate, respiratory rate, response to noxious stimulation, movement, and ambulation every 15 minutes. Heart and respiratory rates were determined by auscultation. Response to noxious stimulation was determined by a negative toe pinch response. Urinary bladder palpation and expression, if needed, were performed every 15 minutes in the male mice and every 60 minutes in the female mice. Urethral plugs, if noted in the distal urethra, were manually extracted. Induction time, anesthetic duration, heart rate, respiratory rate, and the time for return of movement, ambulation, and righting ability were recorded. Induction time was defined, for the purpose of this paper, as the time from anesthetic injection to an absent response to noxious stimulation. Anesthetic duration was defined as the period of time without a response to noxious stimulation after anesthetic induction with survival to anesthetic recovery. Anesthetic recovery was defined as the time for movement, ambulation, and righting ability to return from the initial injection; we used this definition to keep consistency amongst groups that did not result in an anesthetic level. Mice were given at least 48 hours of recovery between injections. Mice were euthanized at the end of the study period with an intraperitoneal overdose of pentobarbital.

### 3.3. Statistics

Data were analyzed using GraphPad Prism version 6 (San Diego, CA) and Stata/IC 13.1 (College Station, TX). Mice were grouped based on anesthetic drug combinations (DTZ and DTZB) and then subgrouped based on drug dosages. Mixed-effects logistic regression was used to evaluate the effects of anesthetic drug doses on the ability to induce an acceptable level of anesthesia; results are presented as odds ratios and 95% confidence intervals (95% CI). Mixed-effects linear regression was used to compare the effects of anesthetic drug dosages on anesthetic induction, duration, and recovery time, as well as their effects on heart and respiratory rates; results are reported as predicted marginal means and standard errors. Cox proportional hazards regression with robust variance estimation to account for replicate measurements in individuals was used to determine whether the rate of induction varied depending on the anesthetic drug administered; results are reported as hazard ratios (HR) and 95% confidence intervals (95% CI). *p* values < 0.05 were considered statistically significant.

## 4. Results

### 4.1. Outcomes and Anesthetic Monitoring in Female Mice

Female mice (*n* = 23) were assigned to the DTZ and DTZB groups with the intent for six mice in each group, although, due to the high mortality associated with the use of tiletamine-zolazepam above 50 mg/kg, numbers in each group were adjusted. Anesthetic trials for the female mice amounted to 43 in the DTZ group and 54 in the DTZB group, resulting in an average of 4 anesthetic trials per mouse, and data are reported in Tables 2 and 3.

Use of dexmedetomidine and tiletamine-zolazepam (DTZ) achieved anesthesia in 58% (25/43) of the anesthetic trials; there were three reported fatalities within this group (dexmedetomidine 0.6 mg/kg and 0.8 mg/kg with tiletamine-zolazepam 40 mg/kg). A strong association with acceptable induction was noted with increasing the dosage of dexmedetomidine from 0.4 mg/kg to 0.6 mg/kg (OR: 10.5, 95% CI: 1.4–80.8, *p* = 0.024), but the higher dosage of 0.8 mg/kg compared to 0.4 mg/kg was not significantly improved (OR: 2.1, 95% CI: 0.4–12.1, *p* = 0.40).

Anesthetic duration varied from 15 minutes to 165 minutes. Anesthetic duration was not significantly altered by increased dosages of dexmedetomidine and tiletamine-zolazepam. Heart rate was significantly elevated with increased dosages of dexmedetomidine from 0.4 to 0.6 mg/kg (187 to 229 bpm, *p* = 0.041) and from 0.6  to 0.8 mg/kg (229 to 254 bpm, *p* = 0.001). Respiratory rate significantly increased (128 to 145 breaths per minute) with increasing dosages of dexmedetomidine in the tiletamine-zolazepam 20 mg/kg group (*p* < 0.001). However, significant interactions were noted for heart rate and respiratory rate with various dosages of dexmedetomidine and tiletamine-zolazepam. At tiletamine-zolazepam dosages greater than 20 mg/kg, increasing the dosages of dexmedetomidine resulted in a decreasing trend in the respiratory rate.

Use of dexmedetomidine, tiletamine-zolazepam, and butorphanol (DTZB) resulted in an anesthetic occurrence of 91% (48/54) in all groups and 100% (36/36) at dexmedetomidine dosages of 0.2 to 0.6 mg/kg and tiletamine-zolazepam 20 to 40 mg/kg. One fatality was reported at dexmedetomidine 0.6 mg/kg, tiletamine-zolazepam 20 mg/kg, and butorphanol 3 mg/kg. Anesthetic duration ranged from 0 to 270 minutes. Induction time ranged from 0 to 69 minutes and recovery time ranged from 75 minutes to 425 minutes. No significant changes in heart rate or respiratory rate were noted with increasing the dosage of either dexmedetomidine or tiletamine-zolazepam within these groups. Anesthetic duration and recovery time were increased with higher dosages of dexmedetomidine and tiletamine-zolazepam (Table 3). The rate of induction was significantly decreased with increased dosages of tiletamine-zolazepam from 10 to 20 mg/kg (HR = 2.2, 95% CI = 1.6–3.0, *p* < 0.001) and from 20 to 40 mg/kg (HR = 4.6, 95% CI = 2.1–10.1, *p* < 0.001).

Dexmedetomidine and tiletamine-zolazepam at dosages of 0.4 to 0.6 mg/kg and 20 to 40 mg/kg, respectively, were compared to the same dosages with the addition of butorphanol at 3 mg/kg SC. Anesthesia was achieved in 58% (14/24) of mice without butorphanol and in 100% (24/24) of mice that received butorphanol. Induction time ranged from 16 to 90 minutes without butorphanol and 8 to 51 minutes with butorphanol. The rate of induction was significantly decreased with the addition of butorphanol (HR = 4.2, 95% CI = 1.8–9.8, *p* = 0.001). Direct statistical comparison between the groups with butorphanol and without butorphanol was unable to be evaluated, as all animals that received butorphanol achieved anesthesia. The odds ratio of dexmedetomidine at 0.4 mg/kg compared to 0.6 mg/kg for resulting in induction of anesthesia was 42.3 (95% CI: 1.0–1855, *p* = 0.052). Anesthetic duration ranged from 15 to 150 minutes without butorphanol and from 60 to 270 minutes with butorphanol. Recovery time ranged from 125 to 318 minutes without butorphanol and from 124 to 425 minutes with butorphanol.

### 4.2. Outcomes and Adverse Effects in Male Mice

Male mice (*n* = 7) had a total of 27 anesthetic trials in the DTZ group. The two male mice (*n* = 2) receiving only dexmedetomidine had a total of 12 anesthetic trials and male mice (*n* = 4) receiving only tiletamine-zolazepam had a total of 21 anesthetic trials.

**Figure 1:**
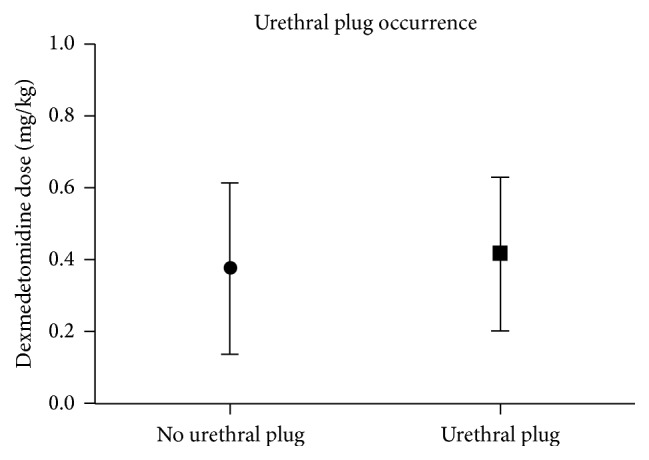
Seminal coagulum plugs occurred at dosages ranging from 0.05 to 0.8 mg/kg. No significant differences were noted in the occurrence of a urethral plug based on the dosage of dexmedetomidine (*p* = 0.61). Data are presented as the mean ± SEM.

**Figure 2:**
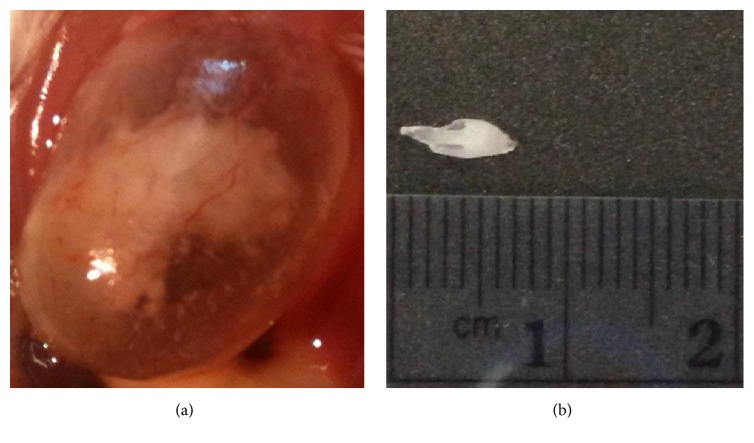
Urinary bladder with homogenous proteinaceous material within it (a) and seminal coagulum plug pulled from the urethra in a male mouse (b).

The incidence of urethral obstructions was 66% (25/38) in male mice that received dexmedetomidine. Use of dexmedetomidine alone resulted in a 67% (8/12) incidence of urethral plugs. When used in combination with tiletamine-zolazepam, dexmedetomidine resulted in a 65% (17/26) incidence of urethral plugs. Tiletamine-zolazepam, when used alone, resulted in a 0% (0/21) incidence of urethral obstructions. The mean dosages of dexmedetomidine were 0.38 mg/kg in the no urethral plug group and 0.42 mg/kg in the urethral plug group, respectively. Increasing the dosage of dexmedetomidine was not associated with an increased occurrence of urethral plugs as shown in Figure 1. Seminal coagulum plugs varied in size from 2 mm to 8  mm. Necropsy confirmed the presence of eosinophilic homogenous proteinaceous material within the urinary bladder and the proximal urethra in one mouse as shown in Figure 2. No bacteria or inflammatory cells were noted within the plug, or within a seminal plug submitted separately from a second mouse.

### 4.3. Mortality

Mortality due to anesthetic complications was 17.4% (4/23) in female mice and 15.4% (2/13) in male mice, resulting in an overall mortality rate from anesthetic complications of 16.7% (6/36) in males and females. Mortality rates, when calculated per anesthetic trial, gave an overall anesthetic mortality of 4.8% (6/124) in all anesthetic trials. Mortality increased with tiletamine-zolazepam at dosages of 50 mg/kg (*n* = 1) and 80 mg/kg (*n* = 1) as well as combinations of dexmedetomidine and tiletamine-zolazepam at 0.6 mg/kg and 40 mg/kg (*n* = 1) and 0.8 mg/kg and 40 mg/kg (*n* = 2) and dexmedetomidine 0.6 mg/kg, tiletamine-zolazepam 20 mg/kg, and butorphanol 3 mg/kg (*n* = 1). The mortality rate due to urethral obstructions was 38% (5/13). Recommended dosages are reported in Table 4 and the drugs used at these dosages had a 0% (0/36) mortality rate. Use of tiletamine-zolazepam at dosages of 40 mg/kg resulted in a 0% (0/36) mortality rate when used in combination with dexmedetomidine at dosages less than or equal to 0.4 mg/kg.

## 5. Discussion

Dexmedetomidine and tiletamine-zolazepam, when used in combination, provided inconsistent levels of anesthesia. The addition of an opioid such as butorphanol resulted in adequate to excellent levels of anesthesia at appropriate dosages. Sedation of male mice with dexmedetomidine resulted in a 66% incidence of urethral obstructions and a 38% mortality rate due to urethral obstructions.

Use of dexmedetomidine and tiletamine-zolazepam was poor at inducing a reliable plane of anesthesia and increased dosages resulted in a higher mortality rate. Induction was faster when the dosage of dexmedetomidine was increased from 0.4 to 0.6 mg/kg, but a ceiling affect was reached at dosages above 0.6 mg/kg. Anesthetic duration was not significantly altered by increased dosages of dexmedetomidine or tiletamine-zolazepam, suggesting that a ceiling effect was reached with these two anesthetic drugs with no benefit of increasing dosages above 0.6 mg/kg of dexmedetomidine or 40 mg/kg of tiletamine-zolazepam. Without the addition of an opioid, the combination of dexmedetomidine and tiletamine-zolazepam cannot be recommended as an anesthetic protocol. This was an unexpected occurrence as the dissociative anesthetic (tiletamine-zolazepam) is thought to be a reliable anesthetic drug in multiple mammalian species. We theorize that in BALB/c mice the combination of these two drugs creates a narrow therapeutic index with the toxic dose in some individuals being lower than the anesthetic dose.

The addition of butorphanol resulted in a 100% (36/36) occurrence of anesthesia at dosages of 0.2 to 0.6 mg/kg of dexmedetomidine and 20 to 40 mg/kg of tiletamine-zolazepam, indicating that the addition of an opioid resulted in a consistent plane of anesthesia at this dose range. Induction time was significantly decreased with increasing dosages of tiletamine-zolazepam, whereas anesthetic duration and recovery time were significantly increased with increased dosages of dexmedetomidine and tiletamine-zolazepam. Direct comparison of the addition of butorphanol was achieved at dexmedetomidine dosages of 0.4 to 0.6 mg/kg with tiletamine-zolazepam dosages of 20 to 40 mg/kg, respectively. Anesthesia was achieved in all groups with the addition of butorphanol and only 58% (14/24) of groups without butorphanol, indicating the need for an opioid to achieve a reliable plane of anesthesia. This is likely due to the addition of a drug with sedative effects at a different receptor site (kappa agonist) potentiating the effect of dexmedetomidine and tiletamine-zolazepam thereby lowering the amount needed for anesthesia to below the toxic dose. Recommended dosages of dexmedetomidine, tiletamine-zolazepam, and butorphanol for use in BALB/c mice are expressed in Table 4. Although the stated dosages resulted in 0% mortality and no complications upon recovery, it is recommended that supplemental oxygen and fluids for hemodynamic support be used to offset the adverse effects of these drugs.

Adverse effects of anesthetic regimens are major concerns, with the most commonly reported adverse effects being respiratory depression and hypoxia [13]. Respiratory depression occurred with tiletamine-zolazepam dosages above 20 mg/kg as dosages of dexmedetomidine were increased, supporting the use of supplemental oxygen with this anesthetic combination. Results appear consistent with other pilot studies in our lab and a previous study involving the use of dexmedetomidine in animals [13].

The occurrence of urethral obstructions appears to be associated with the use of dexmedetomidine, as urethral obstructions occurred in 67% of male mice that received only dexmedetomidine in contrast with 0% of the male mice that received only tiletamine-zolazepam. The incidence of urethral plugs does not appear to be dose related as no significant differences were noted between the individual dosages. Necropsy and analysis of the urethral plug confirmed that the plug was composed of proteinaceous debris; however, the etiology for the development of the urethral plug remains unknown. Seminal plugs have been discovered in male mice postmortem, but, to our knowledge, the etiology of these plugs is also unknown. Urethral obstructions due to seminal coagulum plugs have been reported with the use of medetomidine and ketamine in male mice (C57BL/6 and mixed genetic mice) [12]. Wells et al. discovered that the morbidity and mortality rates were significantly higher (*p* < 0.001) in the group treated with medetomidine-ketamine than in the xylazine-ketamine group [12]. None of the male mice anesthetized with ketamine-xylazine developed urethral obstructions, which suggests that the urethral obstructions were related to the use of medetomidine in this study [12].

As this and previous studies show an association of urethral plugs due to the use of an alpha_2_ agonist, it is reasonable to make the assumption that this is due to effects at either the alpha_1_ or the alpha_2_ receptor. Dexmedetomidine at higher concentrations has been reported to have dual alpha_2_ agonist and alpha_1_ adrenergic antagonist effects [14] while xylazine, a different alpha_2_ agonist which has higher receptor affinity for the alpha_1_ receptor, has not been associated with seminal coagulum plugs [12]. Given that the incidence of urethral obstructions in this study is not dose dependent with dexmedetomidine, this would lead us to believe that the alpha_1_ receptor cannot be the only receptor involved. Given this, the cause of the obstruction is likely multifactorial with the alpha_2_ adrenergic effects of dexmedetomidine resulting in a decreased ability to void the urinary bladder and the alpha_1_ adrenergic effects causing urethral sphincter tightening [15]. At this time, the role of seminal coagulum plugs in this obstructive process is unclear.

Several limitations of this study need mentioning. Due to the experimental design of this study with mice receiving multiple dosages of drugs and our inability to measure drug levels during these experiments, there may have been an additive effect of repeat anesthetic dosages that affected the results although there was a washout period. Groups of mice receiving either dexmedetomidine (*n* = 2) or tiletamine-zolazepam (*n* = 4) alone were small. Increasing the number of mice within each group and therefore the statistical power may have altered the results. Mortality due to anesthetic complications was suspected, but not confirmed, as necropsy was not performed on each of these mice. Only BALB/c mice were studied with these drug combinations; it is likely that the anesthetic duration and recovery times will be altered in mice of different strains. Dosages were lowered from the dexmedetomidine and tiletamine-zolazepam group compared to the dexmedetomidine, tiletamine-zolazepam, and butorphanol group, which limited our ability to directly compare results between these groups. Thus, only a direct comparison of four dosages instead of nine dosages was performed.

In conclusion, the combination use of dexmedetomidine, tiletamine-zolazepam, and butorphanol results in a longer and more reliable duration of anesthesia than the use of dexmedetomidine and tiletamine-zolazepam alone. Research into alternative anesthetic protocols for nonterminal procedures in male mice is needed due to the occurence of urethral obstructions with the use of dexmedetomidine.

## Figures and Tables

**Table 1:** Anesthetic combinations of dexmedetomidine and tiletamine-zolazepam with or without butorphanol. BALB/c female mice were assigned with the intent for six mice in each group, but, due to the high mortality associated with the use of tiletamine-zolazepam above 50 mg/kg, groups were reduced. Dexmedetomidine and tiletamine-zolazepam were given IP and butorphanol was given SC.

Dexmedetomidine (mg/kg)			Dexmedetomidine (mg/kg)		
Tiletamine-zolazepam (mg/kg)			Tiletamine-zolazepam (mg/kg)		
(*n*)			Butorphanol (3 mg/kg)		
			(*n*)		
0.4	0.4	0.4	0.2	0.2	0.2
20	40	60	10	20	40
(*n* = 6)	(*n* = 6)	(*n* = 3)	(*n* = 6)	(*n* = 6)	(*n* = 6)

0.6	0.6	0.6	0.4	0.4	0.4
20	40	60	10	20	40
(*n* = 6)	(*n* = 6)	(*n* = 3)	(*n* = 6)	(*n* = 6)	(*n* = 6)

0.8	0.8	0.8	0.6	0.6	0.6
20	40	60	10	20	40
(*n* = 6)	(*n* = 5)	(*n* = 2)	(*n* = 6)	(*n* = 6)	(*n* = 6)

**Table 2:** Trials with dexmedetomidine and tiletamine-zolazepam (DTZ). Dexmedetomidine and tiletamine-zolazepam were administered IP and butorphanol was administered at 3 mg/kg SC. Data is reported in marginal means and standard error.

	Anesthetic duration (minutes)	Recovery time (minutes)	Anesthesia achieved (%)	Heart rate (bpm)	Respiratory rate (bpm)
Dexmedetomidine 0.4 mg/kg					
Tiletamine-zolazepam					
20 mg/kg	63 (±22)	279 (±25)	50% (3/6)	187 (±14)	128 (±3)
40 mg/kg	67 (±22)	240 (±25)	33% (2/6)	222 (±14)	136 (±3)
60 mg/kg	77 (±22)	240 (±25)	33% (1/3)	227 (±20)	135 (±4)

Dexmedetomidine 0.6 mg/kg					
Tiletamine-zolazepam					
20 mg/kg	23 (±16)	222 (±17)	67% (4/6)	229 (±14)	134 (±3)
40 mg/kg	73 (±13)	265 (±16)	100% (6/6)	209 (±14)	132 (±3)
60 mg/kg	47 (±22)	243 (±25)	67% (2/3)	193 (±20)	130 (±4)

Dexmedetomidine 0.8 mg/kg					
Tiletamine-zolazepam					
20 mg/kg	58 (±18)	253 (±20)	50% (3/6)	254 (±14)	145 (±3)
40 mg/kg	48 (±18)	286 (±35)	40% (2/5)	207 (±16)	130 (±3)
60 mg/kg	175 (±31)	404 (±35)	50% (1/2)	233 (±25)	125 (±5)

**Table 3:** Trials with dexmedetomidine, tiletamine-zolazepam, and butorphanol (DTZB). Dexmedetomidine and tiletamine-zolazepam were administered IP and butorphanol was administered at 3 mg/kg SC. Data is reported in marginal means and standard error.

	Anesthetic duration (minutes)	Recovery time (minutes)	Anesthesia achieved (%)	Heart rate (bpm)	Respiratory rate (bpm)
Dexmedetomidine 0.2 mg/kg					
Tiletamine-zolazepam					
10 mg/kg	28 (±21)	105 (±16)	50% (3/6)	188 (±12)	132 (±4)
20 mg/kg	73 (±16)	168 (±16)	100% (6/6)	191 (±12)	137 (±4)
40 mg/kg	143 (±16)	217 (±16)	100% (6/6)	198 (±12)	137 (±4)

Dexmedetomidine 0.4 mg/kg					
Tiletamine-zolazepam					
10 mg/kg	90 (±18)	161 (±16)	33% (2/6)	199 (±12)	129 (±4)
20 mg/kg	110 (±16)	224 (±16)	100% (6/6)	202 (±12)	134 (±4)
40 mg/kg	163 (±16)	273 (±16)	100% (6/6)	209 (±12)	134 (±4)

Dexmedetomidine 0.6 mg/kg					
Tiletamine-zolazepam					
10 mg/kg	80 (±16)	213 (±16)	100% (6/6)	195 (±12)	131 (±4)
20 mg/kg	195 (17)	276 (±17)	100% (6/6)	198 (±12)	136 (±4)
40 mg/kg	216 (±16)	325 (±16)	100% (6/6)	205 (±12)	136 (±4)

**Table 4:** Recommended dosages of dexmedetomidine, tiletamine-zolazepam, and butorphanol. Dosages recommended for use were determined based on anesthetic duration, induction, and recovery time, as well as safety, as measured by mortality. Increased dosages of dexmedetomidine and tiletamine-zolazepam resulted in an increasing trend of anesthetic duration and recovery times. Dexmedetomidine and tiletamine-zolazepam were administered IP and butorphanol was administered in all above listed trials at 3 mg/kg SC. Data are reported in predicted marginal means and standard error.

	Anesthetic duration (minutes)	Recovery time (minutes)	Anesthesia achieved (%)	Heart rate (bpm)	Respiratory rate (bpm)
Dexmedetomidine 0.2 mg/kg					
Tiletamine-zolazepam 20 mg/kg	73 (±16)	168 (±16)	100% (6/6)	191 (±12)	137 (±4)
Tiletamine-zolazepam 40 mg/kg	143 (±16)	217 (±16)	100% (6/6)	198 (±12)	137 (±4)

Dexmedetomidine 0.4 mg/kg					
Tiletamine-zolazepam 20 mg/kg	110 (±16)	224 (±16)	100% (6/6)	202 (±12)	134 (±4)
Tiletamine-zolazepam 40 mg/kg	163 (±16)	273 (±16)	100% (6/6)	209 (±12)	134 (±4)

## Abbreviations

DTZ: Dexmedetomidine and tiletamine-zolazepam anesthesia group
DTZB: Dexmedetomidine, tiletamine-zolazepam, and butorphanol anesthesia group
IACUC: Institutional Animal Care and Use Committee
AAALAC: Association for Assessment and Accreditation of Laboratory Animal Care.

## References

[1] Alves H. C., Valentim A. M., Olsson I. A. S., Antunes L. M. (2009). Intraperitoneal anaesthesia with propofol, medetomidine and fentanyl in mice. *Laboratory Animals*.

[2] Arras M., Autenried P., Rettich A., Spaeni D., Rülicke T. (2001). Optimization of intraperitoneal injection anesthesia in mice: drugs, dosages, adverse effects, and anesthesia depth. *Comparative Medicine*.

[3] Burnside W. M., Flecknell P. A., Cameron A. I., Thomas A. A. (2013). A comparison of medetomidine and its active enantiomer dexmedetomidine when administered with ketamine in mice. *BMC Veterinary Research*.

[4] Gargiulo S., Greco A., Gramanzini M. (2012). Mice anesthesia, analgesia, and care, Part I: anesthetic considerations in preclinical research. *ILAR journal*.

[5] Gargiulo S., Greco A., Gramanzini M. (2012). Mice anesthesia, analgesia, and care, Part II: anesthetic considerations in preclinical imaging studies. *ILAR Journal*.

[6] He S., Atkinson C., Qiao F., Chen X., Tomlinson S. (2010). Ketamine-xylazine-acepromazine compared with isoflurane for anesthesia during liver transplantation in rodents. *Journal of the American Association for Laboratory Animal Science*.

[7] Izer J. M., Whitcomb T. L., Wilson R. P. (2014). Atipamezole reverses ketamine-dexmedetomidine anesthesia without altering the antinociceptive effects of butorphanol and buprenorphine in female C57BL/6J mice. *Journal of the American Association for Laboratory Animal Science*.

[8] Janssen B. J. A., De Celle T., Debets J. J. M., Brouns A. E., Callahan M. F., Smith T. L. (2004). Effects of anesthetics on systemic hemodynamics in mice. *American Journal of Physiology—Heart and Circulatory Physiology*.

[9] Wenger S. (2012). Anesthesia and analgesia in rabbits and rodents. *Journal of Exotic Pet Medicine*.

[10] Dickerson D. M. (2014). Acute pain management. *Anesthesiology Clinics*.

[11] Sinclair M. D. (2003). A review of the physiological effects of *α*_2_-agonists related to the clinical use of medetomidine in small animal practice. *Canadian Veterinary Journal*.

[12] Wells S., Trower C., Hough T. A., Stewart M., Cheeseman M. T. (2009). Urethral obstruction by seminal coagulum is associated with medetomidine-ketamine anesthesia in male mice on C57BL/6J and mixed genetic backgrounds. *Journal of the American Association for Laboratory Animal Science*.

[13] Ko J. C. H., Abbo L. A., Weil A. B., Johnson B. M., Payton M. (2007). A comparison of anesthetic and cardiorespiratory effects of tiletamine-zolazepam-butorphanol and tiletamine-zolazepam-butorphanol-medetomidine in cats. *Veterinary Therapeutics*.

[14] Hamasaki J., Tsuneyoshi I., Katai R., Hidaka T., Boyle W. A., Kanmura Y. (2002). Dual *α*2-adrenergic agonist and *α*1-adrenergic antagonist actions of dexmedetomidine on human isolated endothelium-denuded gastroepiploic arteries. *Anesthesia and Analgesia*.

[15] Streng T., Santti R., Andersson K.-E. (2010). Voiding effects mediated by *α*2-adrenoceptors in the anaesthetized male rat. *BJU International*.

